# Microstate-based Neurofeedback in Attention Deficit Hyperactivity Disorder Population: A Randomized Controlled Crossover Trial

**DOI:** 10.1007/s10548-025-01161-8

**Published:** 2025-12-01

**Authors:** Victor Férat, Marie-Pierre Deiber, Roland Hasler, Abele Michela, Christoph M. Michel, Nader Perroud, Tomas Ros

**Affiliations:** 1 Functional Brain Mapping Laboratory, Department of Basic Neurosciences, University of Geneva, Geneva, Switzerland; 2 Department of Psychiatry, Faculty of Medicine, University of Geneva, Geneva, Switzerland; 3 Department of Psychiatry, University Hospitals of Geneva, Geneva, Switzerland; 4 Center for Biomedical Imaging (CIBM), Lausanne, Geneva, Switzerland; 5 Department of Psychiatry, Dalhousie University, Nova Scotia, Halifax, NS Canada

**Keywords:** EEG, Neurofeedback, Microstates, ADHD, Psychiatric disorders

## Abstract

Neurofeedback (NFB) therapy based on spectral neuromarkers of Attention-Deficit/Hyperactivity Disorder (ADHD) has faced challenges regarding its efficacy and replicability. In this study, we investigate whether a microstate-based EEG marker, recently implicated in ADHD, could serve as a novel target for neurofeedback. Emerging research suggests that ADHD patients often exhibit an excess of microstate D, a state characterized by fronto-central cortical activity linked to attentional functions. This study aims to assess whether neurofeedback training can effectively modulate microstate D in adult ADHD patients, along with its short-term neurobehavioral correlates. We employed a within-subject, crossover design with 19 adults with ADHD, who participated in two counterbalanced neurofeedback sessions: one aimed at upregulating microstate D percent time coverage, and the other at downregulating it. While patients were able to volitionally increase microstate D during the upregulation session, no significant change was observed during the downregulation session. Direct comparison between the two sessions revealed that online control of microstate D was specific to the closed-loop feedback, rather than merely task engagement. No short-term effects of the neurofeedback sessions were observed. No moderate nor major adverse effects were reported. Despite lack of statistical power, this study provides controlled indicationfor the specificity and safety of neurofeedback training based on microstate D in adult ADHD patients. Although the short-term design did not yield clinical improvements, the findings demonstrate the feasibility of microstate-based neurofeedback protocols in a clinical population and offer valuable technical and methodological insights for designing futur studies.

**Supplementary Information:**

The online version contains supplementary material available at 10.1007/s10548-025-01161-8.

## Introduction

In clinical settings, the use of electroencephalography (EEG) has revealed numerous markers able to identify abnormal neurological activity, associated with various pathologies. Building upon these findings as well as the affordable and non-invasive EEG technique, multiple research groups have used EEG to bridge the gap between research and clinical intervention through the implementation of neurofeedback training (Arns et al. 2017; Ros et al. 2014). Neurofeedback is a biofeedback technique designed to enable self-regulation of (abnormal) biomarkers (Sitaram et al. 2017). By recording brain activity and providing continuous feedback of one’s current brain parameter, neurofeedback protocols enable people to implement strategies to influence their own brain parameters. This closed-loop system can be used to explore the relationship between brain-related metrics, cognitive functions and clinical conditions.

A significant part of the neurofeedback literature has focused on attention deficit hyperactivity disorder (ADHD) (Enriquez-Geppert et al. 2019), a neuro-developmental disorder affecting approximately 5% of children (Polanczyk et al. 2007; Sayal et al. 2018), with more than half continuing to experience it into adulthood (3.1% incidence in adult population (Amiri and Ghoreishizadeh 2014; Fayyad et al. 2016)). ADHD is characterized by persistent patterns of inattention, hyperactivity and impulsivity which can significantly affect daily functioning and overall quality of life (American Psychiatric American, 2013).

Current first-line treatments, involving medication intake (Chaplin 2018; Kooij et al. 2010) show efficacy. However, they are often associated with side effects and have several contraindications that make patients reluctant to use them (Wilens et al. 2011).

For these reasons, neurofeedback has often been tested as an alternative or a complement to pharmacological treatment for ADHD. For the most part, these neurofeedback protocols rely on spectral brain parameters, which measure frequency power at one or more locations on the scalp. In the case of individuals with ADHD, several parameters such as alpha, theta, beta rhythms, and to a further extent the theta/beta ratio, have been extensively used as target measures in neurofeedback protocols (Lubar and Shouse 1976). However, recent discussions (Arns et al. 2018; Barth et al. 2021; Bink et al. 2016; Duric et al. 2017; Schönenberg et al. 2017; Steiner et al. 2011) supported by meta-analyses (Arns et al. 2013) have questioned the reliability of these markers and to some extent the relevance of their respective neurofeedback protocols as new clinical tools for managing ADHD.

With the objective of developing alternative markers, Férat et al. (Férat et al. 2021) have proposed the use of EEG microstates analysis to study ADHD populations. Introduced by Dr. Lehmann in 1972 (Lehmann 1971), this method involves segmenting brain activity into spatially defined states. EEG microstates analysis has the benefit of using the full spatial resolution of high-density EEG recordings, unlike classical spectral approaches which often overlook the spatial component by limiting their analyses to one or a subset of EEG channels. Using two independent datasets, Férat et al. (Férat et al. 2021) revealed abnormal levels of some EEG microstate parameters in individuals with ADHD compared to a healthy population. Specifically, ADHD individuals showed increased levels of a fronto-central state, commonly referred to as microstate D. This state has been repeatedly linked to attentional (dys)functions of the brain (Britz et al. 2010; Custo et al. 2017; D’Croz-Baron et al. 2021; Murphy et al. 2018; Tarailis et al. 2024).

Based on these results, this study aims to validate the feasibility and effectiveness of using microstate-based neurofeedback protocols in ADHD populations. First, we verified whether individuals with ADHD can achieve control over microstate D presence using neurofeedback. Secondly, we verified that such control is specific to the neurofeedback paradigm.

To test this hypothesis, we developed a novel neurofeedback training protocol that directly targets microstate parameters. During the present crossover study, adult ADHD participants were asked to perform two 30-minute microstate-based neurofeedback training sessions in addition to a battery of clinical questionnaires and behavioral tests. The two sessions were identical except for the direction of neurofeedback control, which was different for each session. The order of the sessions was randomized to compensate for any potential learning effect.

The primary endpoint of this study was to evaluate changes in the time coverage of microstate D across distinct phases of neurofeedback training, both during up-regulation and down-regulation sessions, as well as the between-session contrasts. The secondary outcomes include assessing participants’ performance on attentional tasks to determine the impact of neurofeedback on cognitive function, and analyzing changes in clinical questionnaire scores, which offer further insights into how neurofeedback influences psychological and clinical markers.

## Methods

A version of the CRED-nf checklist (Ros et al. 2020) is provided (supplementary Table 1) to help readers navigate this method section.

### Sample Size

Estimates of clinically relevant effect sizes were derived from studies by Férat et al. (Férat et al. 2021) which reported effect sizes of d = 0.56 and d = 0.81 for differences in microstate D duration between ADHD and control groups, and from a feasibility study on microstate-neurofeedback in a healthy population (Hernandez et al. 2015), which reported an effect size of d = 1.0 for increases in microstate D duration during neurofeedback training. Based on these estimates, we considered the worst-case scenario with an effect size of d = 0.56. To achieve a power of 80% with α = 0.05 using a one-sample t-test, the expected outcome requires a sample size of 21 participants.

### Participants

20 ADHD patients were recruited through the Adult ADHD Unit at Geneva University Hospitals from September 2022 to April 2023. One participant was excluded after the clinical screening session due to the discovery of an exclusion criterion. The final sample therefore consisted of 19 patients (7 females, mean age: 30.26, SD: 8.27), divided into two ADHD subtypes: the “mixed” one composed of 10 patients and the “inattentive” one composed of 9 patients. Participants received a voucher worth CHF 300 to be used in the local shop if they took part in the study in full. This study was approved by the Research Ethics Committee of the Republic and the Canton of Geneva [project number 2022 − 00848] and conducted in accordance with the principles embodied in the Declaration of Helsinki and in accordance with local statutory requirements. All participants gave written informed consent to participate in the study. The trial was terminated prematurely due to the conclusion of the funding period.

This study was registered at ClinicalTrials.gov (NCT05582928).

**Fig. 1:**
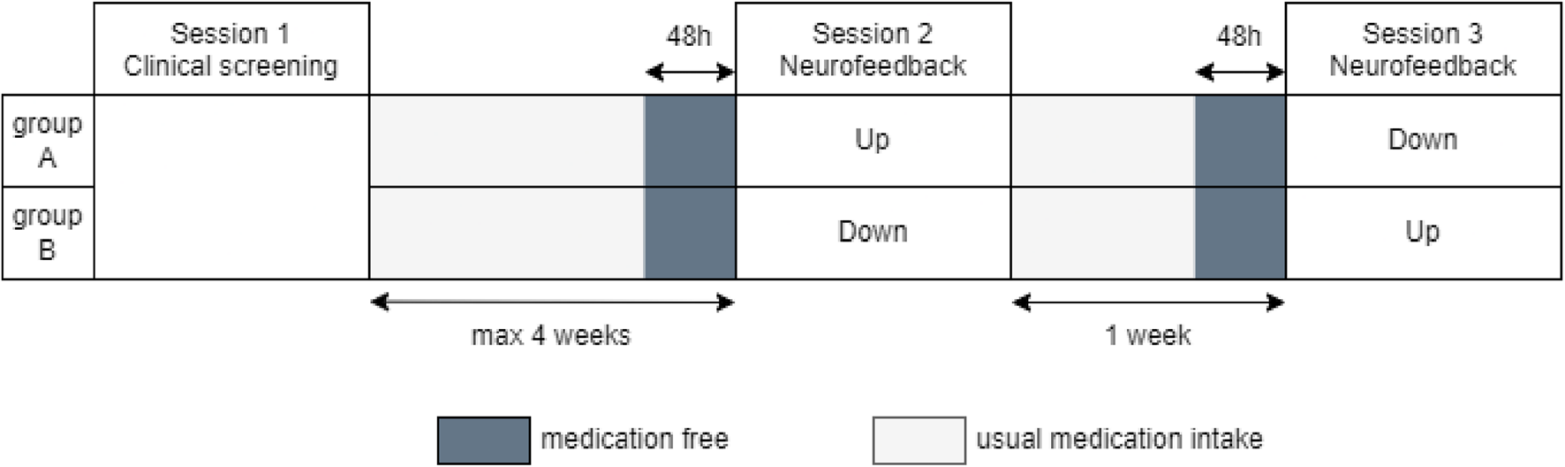
Experimental design: After a clinical screening session, participants were randomly assigned to two experimental groups. Both groups underwent two microstates neurofeedback sessions at a one-week interval, but while group A started with the up-regulation of microstate D, group B started with the down-regulation. A 48-hour drug-free period was ensured before each neurofeedback session to minimize the effect of treatments

### Experimental Design

The experimental design (Fig. 1) included three sessions: a clinical screening session (session 1) and two neurofeedback sessions (session 2 and 3).

To minimize the learning effect across sessions, each participant was assigned to a group: group A was asked to increase their microstate parameter during session 2 and to decrease the same parameter in session 3. Conversely, group B was asked to decrease their microstate parameter during session 2 and to increase it in session 3. Group assignment was conducted on a rolling basis, determined by participants’ recruitment order.

Moderate adverse events, such as significant discomfort, persistent headaches, or emotional distress requiring intervention and severe adverse events, defined as any outcomes requiring medical attention or resulting in lasting harm (regardless of their relation to the study) were systematically monitored throughout the entire enrollment period.

### Medication

To control for the effect of medication while minimally interfering with patients’ treatments, participants were asked to stop psychostimulant intake at least 48 h before each neurofeedback session.

#### Session 1: Clinical Screening Session

Session 1 consisted of a clinical screening interview to assess participants’ current symptoms of ADHD. After giving their informed written consent, patients underwent four clinical questionnaires, including the Adult ADHD Self-Report Scale (ASRS v1.1) (Kessler et al. 2005) which evaluates current ADHD symptoms in 18 questions in adolescents and adults. The clinician’s diagnosis was based on three structured questionnaires: ADHD Child Evaluation for Adults (ACE+) (https://www.psychologyservices.uk.com/adhd.htm), the French version of the Structured Clinical Interview for DSM-IV Axis II Personality Disorders (SCID-II) (Gorgens 2011) and the French version of the Diagnostic Interview for Genetic Studies (mood disorder part only) (Nurnberger et al. 1994; Preisig et al. 1999).

**Fig. 2:**
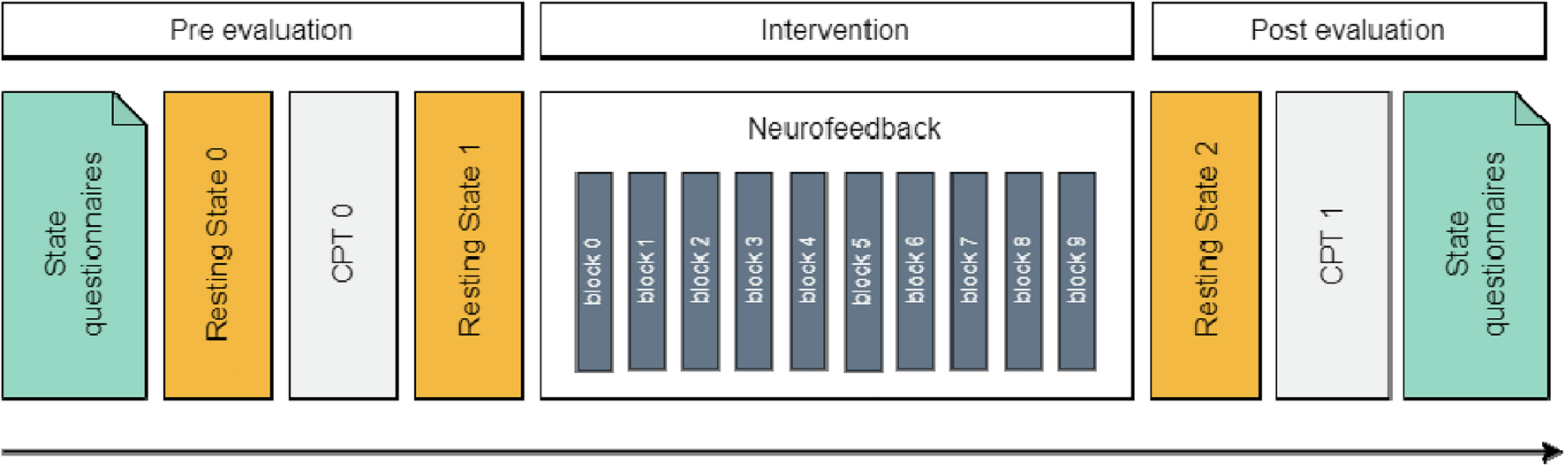
Session design: Effect of the microstate neurofeedback intervention was evaluated using pre and post evaluation which included clinical questionnaires, resting state recordings as well as continuous performance tasks (CPT)

#### Session 2 & 3: EEG Neurofeedback

Neurofeedback sessions (session 2 and session 3) were composed of three consecutive parts (Fig. 2):

Pre-evaluation: Evaluation of the participant’s current state using standard clinical questionnaires (State Related Questionnaire pre), an EEG recording at rest (resting state 0), a continuous performance task (CPT pre), and another EEG recording at rest (resting state 1).

Neurofeedback: 10 successive identical blocks composed of 3 min of neurofeedback training interleaved with 20 s breaks.

Post-evaluation: Evaluation of current participant state by means of an EEG recording at rest (resting state 2), a continuous performance task (CPT post), and standard clinical questionnaires (State-Related Questionnaire post).

### State Clinical Questionnaires

Clinical questionnaires were used to assess the current psychological state at the beginning and end of each neurofeedback session. Questionnaires included the Positive and Negative Affect Schedule (Tran 2013): a 20-item test using a 5-point scale that ranges from very slight or not at all (1) to extremely (5), as well as the Activation-Deactivation Adjective Check List (Thayer 1986): a 40-item test for rapid assessments of momentary activation or arousal states.

### EEG Recordings

The EEG was continuously recorded throughout sessions 2 and 3 using a 64 Ag/AgCl electrode cap according to the international system of 10–20, with a sampling rate of 500 Hz (ANT Neuro, The Netherlands). The ground electrode was placed on the scalp at a site equidistant between Fpz and Fz, and the reference electrode was placed at CPz. Electrical signals were amplified using the eego mylab system (ANT Neuro, The Netherlands), and all electrode impedances were kept below 5 kΩ. Stimuli and response timestamps were marked in the EEG recording by sending a signal from the computer to the eego mylab system using parallel port signals.

### Continuous Performance Task

A continuous performance task (CPT) was implemented using Psychopy3 (Peirce et al. 2019). This attention/inhibition task consisted of a presentation stream of letters appearing for 200ms. Subjects were instructed to press the space bar on their keyboard as fast as possible when any letter (Go trial) except the target letter X (NoGo trial) appeared. The maximal response window was 600 ms after the letter’s presentation. An inter-trial period of random duration (uniform distribution [800–1000ms]) was used between each trial to minimize prediction effects. In total, the participants underwent 240 tests, with 75% Go tests and 25% NoGo tests for a total duration of approximately 6 min.

### Neurofeedback

#### Calibration

To adapt neurofeedback to the participant’s current situation, the resting state phase preceding neurofeedback (resting state 1) was used as calibration. The 3 min recording was preprocessed as follows: signals were filtered between 1 and 30 Hz band using a Butterworth bandpass zero-phase (two-pass forward and reverse) non-causal filter and notched filtered between 49 and 51 Hz using a one-pass, zero-phase, non-causal band-stop filter. Bad electrodes and artifactual data segments were manually annotated and discarded from further analysis. Independent component analysis (ICA) was computed for a number of 12 components. Components showing eye blinks (strong frontal activity as well as sparse temporal activations), horizontal eye movements (strong positive and negative frontal activity as well as sparse temporal activations), but also muscular components were rejected, and the EEG signal was reconstructed. Continuous recording was split into 3 s epochs with a 0.5 s overlap to mimic neurofeedback processing. Template microstate topographies were backfitted to each epoch using Pycrostates (Férat et al. 2022). No smoothing was applied. For each epoch, the time coverage of microstate D was computed. The minimum and maximum thresholds were computed as follows based on the resulting distribution’s mean (μ) and standard deviation ():

- upper one-standard deviation limit: $max=\mu+\sigma$

- lower one-standard deviation limit: $min=m\pm\sigma$ .

#### The Neurofeedback Loop

The online data was acquired using the brain streaming layer (https://github.com/bsl-tools/bsl) a wrapper around Lab Streaming Layer (LSL). At each iteration, the last 3 s of data were acquired. The mastoids electrodes (M1 and M2) were removed from the analysis. EEG signals were filtered using a Butterworth bandpass zero-phase (two-pass forward and reverse) noncausal filter and notched filtered using a one-pass, zero-phase, noncausal band-stop filter. The ICA solution computed during the calibration step was used to remove non brain components from the signal, then bad channels were interpolated, and EEG was re-referenced to average.

**Fig. 3:**
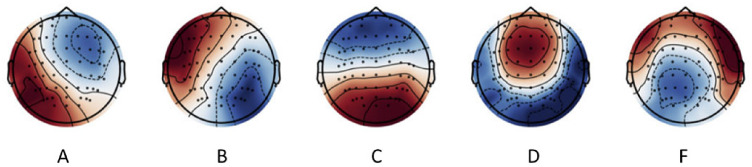
Microstate Templates: The five grand average EEG microstate topographies were obtained from dataset 2 of Férat et al. 2022

The five grand average EEG microstate topographies (Fig. 3) were obtained from dataset 2 of Férat et al. (Férat et al. 2021) and were used as templates for the current study. Template microstates maps were backfitted to the signal without smoothing to estimate microstate D time coverage.

To enhance feedback fluidity and minimize abrupt score fluctuations while maintaining real-time feedback, the score value was calculated as the average of the last 10 microstate D time coverage values linearly weighted by time. Higher weights were given to more recent metrics and lower weights to the least recent ones.

To adapt neurofeedback difficulty to each participant and session, the score value was then linearly scaled to range between − 1 and + 1 using the maximum and minimum values estimated during the calibration step to compute the feedback value according to the formula 1 (supplementary Fig. 1):

1 $$\:\text{feedback}=1-2\cdot\:\frac{\text{max}-{D}_{\text{time coverage}}}{\text{max}-\text{min}}\:$$

Feedback was presented on a 19” screen with a 60 Hz refresh rate.

The feedback consisted of a rectangular gauge placed horizontally in the center of the screen. When the feedback value was negative, the gauge filled linearly to the left, gradually filling completely with a feedback value of −1. Similarly, when the feedback value was positive, the gauge filled to the right, gradually filling completely for a feedback value of 1.

For the up-regulation protocol, the gauge was colored dark blue when the *feedback value* was positive and dark gray for the negative *feedback value*.

For the down-regulation protocol, the gauge was colored dark orange when the *feedback value* was negative and dark gray for the positive *feedback value*.

Each iteration took around 250ms to perform, without any delay between iterations. Overall, considering the window size, the iteration duration and the smoothing procedure, each real-time score could be influenced by data points acquired up to 6.5 s prior.

Participants were not provided with specific instructions regarding the neurofeedback strategy, although they were informed that the study focused on attention. They were only instructed on the direction to move the feedback representation and had no knowledge of how the feedback was calculated and therefore were blinded to the nature of each session’s intervention.

### Analysis

#### Preprocessing

The analysis of EEG recordings was performed using MNE-python (Gramfort 2013). Mastoids electrodes (M1 and M2) were rejected for further analysis. EEG recordings were filtered using a Butterworth bandpass zero phase (two-pass forward and reverse) noncausal filter (filter order 16 (effective, after forward-backward) - Cutoffs at 1.00, 30.00 Hz: −6.02, −6.02 dB) and notched filtered using a one-pass, zero-phase, non-causal band-stop filter with the following parameters:

- Windowed time-domain design (firwin) method.
- Hamming window with 0.0194 passband ripple and 53 dB stopband attenuation.
- Lower passband edge: 49.38.
- Lower transition bandwidth: 0.50 Hz (−6 dB cutoff frequency: 49.12 Hz).
- Upper passband edge: 50.62 Hz.
- Upper transition bandwidth: 0.50 Hz (−6 dB cutoff frequency: 50.88 Hz).
- Filter length: 3381 samples (6.604 s).

Pairs of bridged electrodes were identified using the MNE-python implementation of the intrinsic Hjorth algorithm (based on (Greischar et al. 2004; Tenke and Kayser 2001) and the current implementation of EEGLAB (Delorme and Makeig 2004). EEG time series were then visually inspected, and periods of artifact datapoints, as well as bad channels, were annotated and rejected from further analysis.

Independent component analysis (ICA) was used to remove non-brain-related EEG components. After whitening, principal analysis was used to extract the N largest variance components of the recording, N being equal to the rank of the data. The extended-infomax algorithm (Lee et al. 1999) was then used to calculate the mixing and unmixing matrices of the ICA.

ICA components were visually inspected both in terms of temporal (i.e., time series) and spatial (i.e. topographies). Components that show eye blinks (strong frontal activity as well as sparse temporal activation), horizontal eye movements (strong positive and negative frontal activity as well as sparse temporal activation), as well as muscular components were rejected, and the EEG signal was reconstructed.

For each pair of bridged electrodes, a virtual channel was created midway between the bridged pairs, and bridged electrodes were set as bad. Finally, the bad electrodes were interpolated using spherical spline interpolation (Perrin et al. 1989).

Microstate analysis EEG microstate analysis was performed using Pycrostates (Férat et al. 2022). The five grand average EEG microstate topographies obtained from dataset 2 of Férat et al. (Férat et al. 2021) were used as templates for the current study.

Each time point in the EEG recording was assigned to the topography with which it shared the highest absolute spatial correlation. For offline analysis, an effective smoothing window size of 24 samples (0.0957 s) was used to ensure the temporal continuity of the signal by adjusting the correlation of the central time point with a smoothing factor of 10. Identical label sequences that did not reach a duration of 3 samples (24.0 ms) were split into two parts, each sharing the highest spatial correlation with its neighboring segment and relabeled accordingly.

At the end of this procedure, each EEG recording was accompanied by a discrete sequence of labels that assign each time point to a microstate. From these sequences, three spatio-temporal metrics were computed:

- The global explained variance (GEV) (%) defined as the sum of variances weighted by the global field power of all time points assigned to a label.
- The time coverage (%) defined as the proportion of time during which a label is present in the recording.
- The mean duration (ms), which is the mean temporal duration during which a label is present without interruption. This metric is expressed in milliseconds.

### Statistics

Statistical analysis was performed using the Python version of Dabest v2023.02.14 (Ho et al. 2019). Paired Cohen’s d in microstate D time coverage between control and test conditions were calculated using 5000 bootstrap samples. Confidence intervals were bias corrected and accelerated. P-values of the two-sided permutation t-test were also calculated. The reported values represent the probability of observing the effect size or greater (Cohen’s d), assuming that the null hypothesis of zero difference is true. For each p-value, 5000 reshuffles of the control and test labels were performed.

Within-subject associations of microstate D levels across training runs were examined using repeated-measures correlation.

## Results

After excluding EEG recordings with excessive noise during the experimental procedure, data from *N* = 16 participants were analyzed for the up-regulation session, *N* = 13 for the down-regulation session, and *N* = 12 for the between-session analysis.

**Fig. 4:**
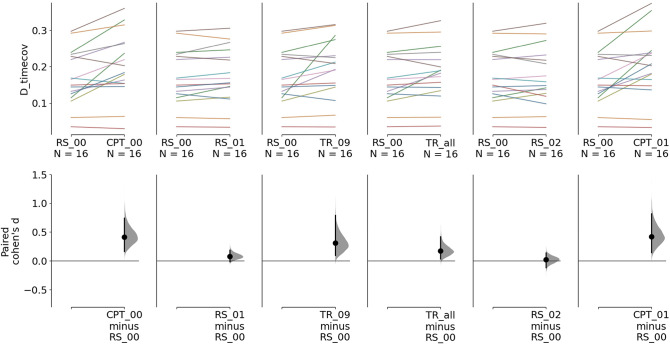
Up regulation session: Estimation plots of paired Cohen’s d in microstate D time coverage between experimental conditions and baseline during the up-regulation session. The first row represents the paired microstate D time coverage values between experimental conditions (RS: resting state, TR: neurofeedback training, CPT: continuous performance task) and baseline (RS_0_). Each line represents a participant. The second row displays the 95% confidence interval (95% CI) estimated through non-parametric bootstrap resampling of the paired Cohen’s d between each experimental condition and baseline

## Primary Outcome Measures

### Up Regulation

#### Neurofeedback

On average, the prevalence of microstate D was also increased during neurofeedback training *TR*_*all*_ compared to baseline (*RS*_0_) (*p* = 0.0404, d = 0.171, 95% CI: [0.0388, 0.418]). A stronger effect was observed when comparing only the last neurofeedback run (*TR*_9_) to baseline (*RS*_0_) (*p* = 0.011, d = 0.304, 95% CI: [0.0994, 0.793]).

#### Resting State

Non-significant increases in microstate D time coverage were observed between *RS*_1_ and *RS*_0_ (*p* = 0.109, d = 0.0775, 95% CI: [−0.0114, 0.188]) and between *RS*_2_ and *RS*_0_ (*p* = 0.733, d = 0.0225, 95% CI: [−0.119, 0.139]).

### Down Regulation

**Fig. 5:**
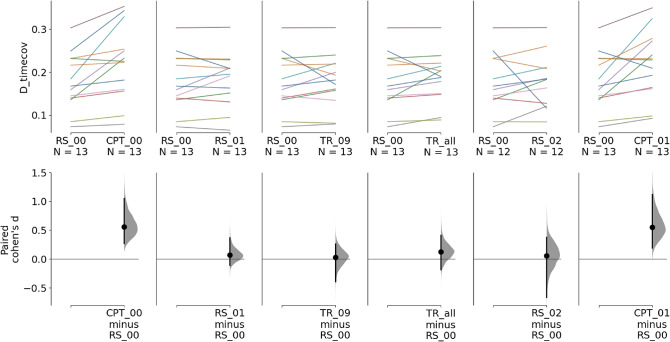
Down regulation session: Estimation plots of paired Cohen’s d in microstate D time coverage between experimental conditions and baseline during the down regulation session. The first row represents the paired microstate D time coverage values between experimental conditions (RS: resting state, TR: neurofeedback training, CPT: continuous performance task) and baseline (RS_0_). Each line represents a participant. The second row displays the 95% confidence interval (95% CI) estimated through non-parametric bootstrap resampling of the paired Cohen’s d between each experimental condition and baseline

#### Neurofeedback

No significant changes in the coverage of microstate D time were observed between neurofeedback runs (*TR*_*all*_: *p* = 0.402, d = 0.123, 95% CI: [−0.184, 0.417], *TR*_9_: *p* = 0.875, d = 0.0256, 95% CI: [−0.389, 0.262] and at baseline (*RS*_0_) during the down-regulation session.

#### Resting State

Similarly, no significant changes in microstate D time coverage were observed between resting states runs (*RS*_1_: *p* = 0.49, d = 0.0721, 95% CI: [−0.109, 0.374], *RS*_2_: *p* = 0.848, d = 0.0535, 95% CI: [−0.665, 0.383] and baseline (*RS*_0_).

### Complementary Analysis

#### Analysis of within-session Learning

**Fig. 6:**
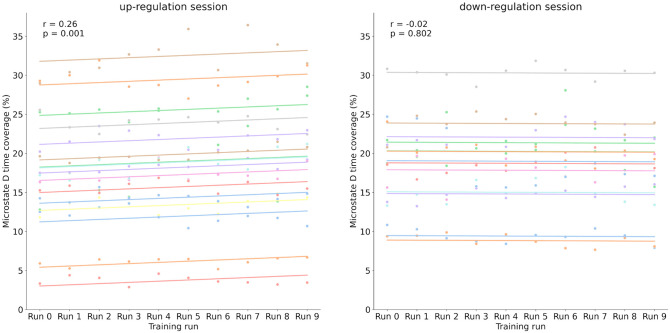
Scatter plots representation of the relationship between training runs (x-axis) and microstate D time coverage (%) (y-axis) for the up (left) and down (right) conditions. Lines indicate fitted linear regression models of each participant. Group level correlation coefficient and p values are reported on each graph. A significant positive association was observed for the up condition (*r* = 0.26, p < 0.001), whereas no significant relationship was found for the down condition (*r* = −0.02, *p* = 0.802)

Linear regression analyses were conducted to examine whether microstate D parameters changed systematically across training runs (supplementary Figs. 2 and 3). As shown in Fig. 6, a significant positive relationship was observed in the *up* condition, indicating that microstate D coverage increased with repeated training (*r = 0.26*,* p 0 0.001*). In contrast, no significant relationship emerged in the *down* condition (*r = −0.02*, *p = 0.802*), suggesting that training runs did not influence effect sizes in this case.

### Continuous Performance Task

During the up-regulation session (Fig. 4), an increase in microstate D time coverage was observed between *CPT*_0_ and *RS*_0_ with an estimated effect size of 0.414 (*p* = 0.0048, 95% CI: [0.167, 0.743]). An increase in microstate D time coverage was observed between *CPT*_1_ and *RS*_0_ with an effect size of 0.423 (*p* = 0.0134, 95% CI: [0.147, 0.819]).For the down-regulation session (Fig. 5), large increases in microstate D time coverage were also observed between *CPT*_0_ (*p* = 0.0048, d = 0.56, 95% CI: [0.269, 1.05]) and *CPT*_1_ (*p* = 0.0048, d = 0.55, 95% CI: [0.193, 1.12]) and baseline (*RS*_0_).

Comparisons of microstate D time coverage during the CPT before (CPT0) and after (CPT_1_) neurofeedback did not yield any significant results for neither the up (two-sided permutation t-test, *p* = 0.80, d = 0.0124, 95% CI: [−0.0975, 0.105]) nor the down (two-sided permutation t-test, *p* = 0.91, d = −0.0385, 95% CI: [− 0.538, 0.171]) regulation session. Up-regulation vs. Down-regulation.

### Baseline Variations

To assess whether any differences were present at baseline between the up- and down-regulation sessions, we compared the relevant measures before neurofeedback training across the two regulation directions. None of these comparisons revealed significant differences. Specifically, microstate D coverage showed no effect (*p* = 0.793, *d* = −0.063, 95% CI [−0.518, 0.306]), attentiveness (*p* = 0.847, *d* = 0.000, 95% CI [−1.090, 0.667]) and active (*p* = 0.748, *d* = 0.196, 95% CI [−0.345, 0.799]) levels remained stable. Similarly, commission errors (*p* = 0.0758, *d* = 0.231, 95% CI [−0.008, 0.553]) nor omission errors (*p* = 0.786, *d* = −0.029, 95% CI [−0.768, 0.513]) significantly differentiated.

#### Microstate Modulation

**Fig. 7:**
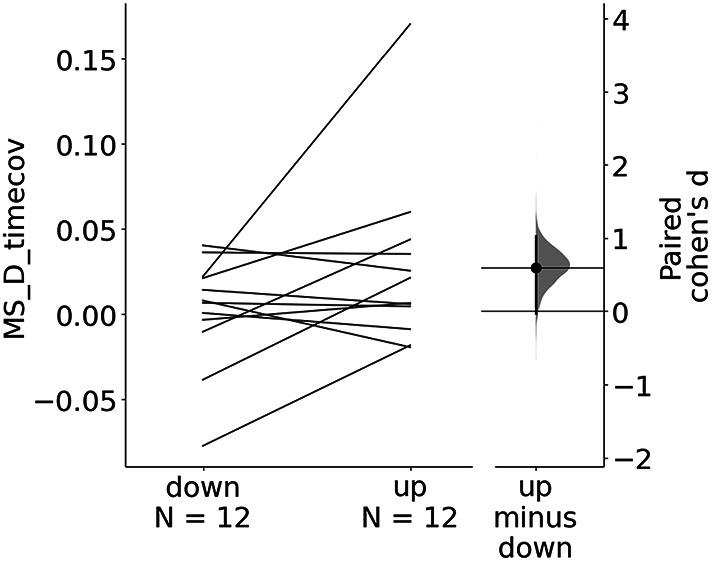
Crossover analysis: Estimation plots of paired Cohen’s d of the difference of microstate D time coverage between the last neurofeedback training block TR9 and baseline RS0 between the up- and down- regulation sessions. The 95% confidence interval (95% CI) estimated through non-parametric bootstrap resampling of the paired Cohen’s d between sessions is displayed next to paired microstate D time coverage differences (TR9 - RS0) of each session

We also evaluated the probability of directly observing a higher effect size of the microstate D time coverage variation (between neurofeedback training and baseline: *TR*_9_ - *RS*_0_) in the up-regulation compared to the down regulation session assuming the null hypothesis of zero difference between the up and down regulation paradigms is true (Fig. 6). A significant increase was observed (one-sided permutation t-test, *p* = 0.043, d = 0.595, 95% CI: [0.0866, 0.951]) (Fig. 7).

### CPT Behavioral Performance

Analyses of CPT behavioral measures did not reveal any significant differences between the up and down regulations sessions both in terms of commission errors (two-sided permutation t-test, *p* = 0.61, d = −0.20, 95% CI: [−1.01, 0.66]) and omission errors (two-sided permutation t-test, *p* = 0.10, d = 0.828, 95% CI: [0.00, 1.640]).

### State Questionnaires

No changes in self-state assessment were observed between the up and down regulation session in terms of Active (two-sided permutation t-test, *p* = 0.096, d = 0.39 95% CI: [−0.13, 0.91]) and Attentive (two-sided permutation t-test, *p* = 0.13, d = 0.54, 95% CI: [−0.09, 1.26]).

### Adverse Events

No moderate or major adverse effects were reported during the trial.

## Discussions

The present study assessed the feasibility of a novel EEG microstate-based neurofeedback protocol in adults with ADHD. While previous work demonstrated microstate modulation in healthy participants (Asai et al., 2022; Hernandez et al. 2015), this is the first application in a clinical population. The primary goal was to determine whether microstate D could be volitionally modulated via feedback and whether this modulation reflected true closed-loop learning rather than general task engagement. Thus, the findings should be viewed as protocol validation, guiding future clinical trials rather than providing evidence of therapeutic efficacy.

A key issue in neurofeedback research is distinguishing genuine self-regulation from nonspecific effects such as task engagement or attentional load. This is particularly relevant for microstate D, consistently associated with attention and cognitive control (Britz et al. 2010; Custo et al. 2017; Murphy et al. 2018; Tarailis et al. 2024). In our study, microstate D increased across all CPT blocks (d = 0.41–0.56), consistent with its link to attentional processes. However, the crossover design allowed direction-specific comparisons, revealing distinct modulation levels in up- versus down-regulation sessions (*p* = 0.043, d = 0.595). Baseline equivalence across sessions supports the conclusion that these effects were training-specific rather than due to general engagement. These findings address a limitation of prior work (Hernandez et al. 2015) which lacked control conditions and therefore could not confirm neurofeedback specificity.

Despite these promising indications, several methodological limitations constrain the interpretation of our findings. Post hoc power analysis indicated that the crossover comparison achieved only 61% power, below the conventional 80% threshold. This reduction can be attributed to two main factors: a smaller-than-planned sample size and lower observed effect sizes. The initial sample size estimation was based on clinically meaningful effects reported in previous studies (d = 0.56–1.0), which were not reached in the present trial. Future studies should therefore plan for small-to-medium effect sizes and consider multi-session training paradigms to enhance the neurofeedback effect and improve statistical power.

The smaller sample size compared with the initial trial registration reflects the practical and technical challenges of using high-density EEG microstate parameters as neurofeedback targets. Data loss stemmed from the technical demands of microstate-based neurofeedback, which requires high-quality, full-scalp EEG recordings that are particularly susceptible to artifacts. Future studies would benefit from real-time preprocessing algorithms capable of managing heavily artifacted data and from incorporating an estimated 20–30% data loss margin during power and sample size planning to accommodate the high technical demands of this method.

We observed evidence of successful volitional control during up-regulation training. Participants demonstrated an increase in microstate D time coverage during neurofeedback compared to baseline, with effect sizes of d = 0.171 for all training blocks combined and d = 0.304 for the final training block (TR9). Although the overall analysis showed limited statistical power (30%) due to small observed effect sizes, a significant within-subject association was found between training block number and microstate D coverage (*r* = 0.26, *p* = 0.001). This progressive improvement across training runs suggests a learning effect, reflecting active participant control rather than the intrinsic cognitive load of performing the task.

Clinically, the demonstration of up-regulation success is not directly relevant for the ADHD population, as individuals with ADHD typically exhibit abnormally elevated microstate D levels at rest (Férat et al. 2021) Nonetheless, these results may hold significant implications for other psychiatric conditions characterized by reduced microstate D presence, such as schizophrenia (Da Cruz et al. 2020; Iftimovici et al. 2023; Nishida et al. 2013).

In contrast, no evidence of successful modulation emerged during down-regulation training. This directional asymmetry suggests that decreasing microstate D may be intrinsically more challenging. Several mechanisms could explain this null result. First, a floor effect may have occurred: given that neurofeedback inherently demands sustained attention, microstate D levels may have already been elevated, limiting our ability to observe a decrease. In addition, down-regulation of an attention-related neural marker may be particularly difficult for individuals with ADHD, whose attentional control is already compromised.

### Future Directions

While the current limitations of this study prevent strong clinical conclusions, they nonetheless provide valuable guidance for future research. Ideally, subsequent studies should adopt multi-session longitudinal designs to assess cumulative learning effects and potential behavioral benefits, as single-session protocols may yield only small effect sizes. Large-scale randomized controlled trials incorporating sham neurofeedback conditions will be essential to establish clinical efficacy. Given the technical challenges inherent to microstate-based neurofeedback, future studies should also account for an expected data loss of 20–40% during study design and power calculations. Furthermore, refined feedback modalities should be implemented to mitigate potential floor effects arising from the intrinsic attentional demands of neurofeedback training.

## Conclusion

In conclusion, this study suggests that microstate D can be specifically modulated through neurofeedback in adults with ADHD. Despite the absence of behavioral transfer or resting-state changes, the feasibility, specificity, and safety results of this exploratory study lay the groundwork for future large-scale clinical trials evaluating the efficacy of microstate-based neurofeedback in clinical populations.

## Supplementary Information

Below is the link to the electronic supplementary material.

Supplementary Material 1

## Data Availability

The data that supports the findings of this study is available from the corresponding author, VF, upon reasonable request.
