# Supplementary material for: Microstate-based Neurofeedback in Attention Deficit Hyperactivity Disorder Population: A Randomized Controlled Crossover Trial
Source: Brain Topogr. 2025 Dec 1;39(1):6. doi: 10.1007/s10548-025-01161-8 (PMC12669258; doi:10.1007/s10548-025-01161-8)
Supplement: Supplementary file 1 — Supplementary Material 1 [file 10548_2025_1161_MOESM1_ESM.docx]

| **Domain** | **Item #** | **Checklist item** | **Reported on page #** |
| --- | --- | --- | --- |
| **Pre-experiment** | | | |
|  | 1a | Pre-register experimental protocol and planned analyses | 9 |
|  | 1b | Justify sample size | 8 |
| **Control groups** | | | |
|  | 2a | Employ control group(s) or control condition(s) | 10 |
|  | 2b | When leveraging experimental designs where a double-blind is possible, use a double-blind | NA |
|  | 2c | Blind those who rate the outcomes, and when possible, the statisticians involved | NA |
|  | 2d | Examine to what extent participants and experimenters remain blinded | NA |
|  | 2e | In clinical efficacy studies, employ a standard-of-care intervention group as a benchmark for improvement | NA |
| **Control measures** | | | |
|  | 3a | Collect data on psychosocial factors | 13 |
|  | 3b | Report whether participants were provided with a strategy | 19 |
|  | 3c | Report the strategies participants used | NA |
|  | 3d | Report methods used for online-data processing and artifact correction | 15 - 19 |
|  | 3e | Report condition and group effects for artifacts | NA |
| **Feedback specifications** | | | |
|  | 4a | Report how the online-feature extraction was defined | 15 - 19 |
|  | 4b | Report and justify the reinforcement schedule | NA |
|  | 4c | Report the feedback modality and content | 18 |
|  | 4d | Collect and report all brain activity variable(s) and/or contrasts used for feedback, as displayed to experimental participants | 18 |
|  | 4e | Report the hardware and software used | 16 |
| **Outcome measures** | | | |
| Brain | 5a | Report neurofeedback regulation success based on the feedback signal | NA |
|  | 5b | Plot within-session and between-session regulation blocks of feedback variable(s), as well as pre-to-post resting baselines or contrasts | 24 - 33 |
|  | 5c | Statistically compare the experimental condition/group to the control condition(s)/group(s) (not only each group to baseline measures) | 24 - 33 |
| Behaviour | 6a | Include measures of clinical or behavioural significance, defined a priori, and describe whether they were reached | NA |
|  | 6b | Run correlational analyses between regulation success and behavioural outcomes | NA |
| **Data storage** | | |  |
|  | 7a | Upload all materials, analysis scripts, code, and raw data used for analyses, as well as final values, to an open access data repository, when feasible | 41 |

***Supplementary table 1****: Consensus on the Reporting and Experimental Design of clinical and cognitive-behavioural Neurofeedback studies (CRED-nf) best practices checklist.*

###
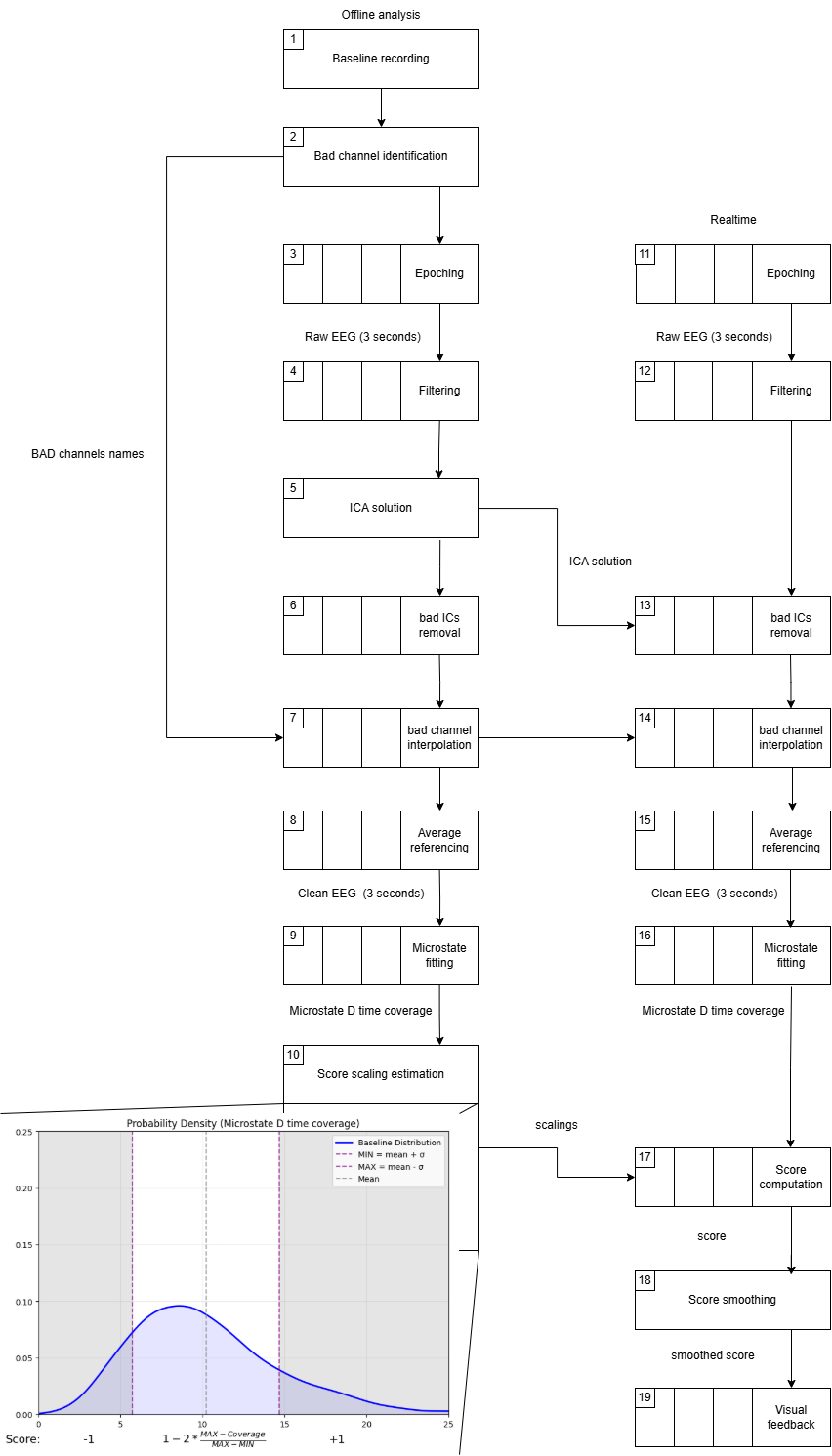


### Supplementary Fig 1. Neurofeedback loop processing pipeline

*An initial offline analysis was performed on the baseline EEG data (1 – 10). First, bad channels were visually identified and removed (2). The data were then segmented into 3-second epochs with 50% overlap to reproduce the real-time data format (3). Each epoch was filtered using a zero-phase Butterworth bandpass filter and a notch filter (4). An independent component analysis (ICA) solution was computed using all epochs (5), then artifactual components were excluded from the data (6). Previously identified bad channels were then interpolated (7) and cleaned signal was subsequently re-referenced to the average (8). Microstate segmentation was then applied to the artifact-free EEG to derive the microstate D time coverage. The coverage values of all baseline epochs were used to construct a distribution from which the score-scaling parameters (minimum and maximum values) were estimated and stored for normalizing subsequent real-time feedback computation.*

*During neurofeedback training (11 – 19), the real-time neurofeedback loop operated continuously throughout each experimental session. At each iteration, a 3-second data window of raw EEG was acquired (11). The signal then underwent the same preprocessing steps — filtering (12), ICA-based artifact removal (13), bad-channel interpolation (14), and average re-referencing (15) — using the ICA solution derived during offline calibration (5). The resulting clean EEG was backfitted to the predefined microstate templates to estimate the ongoing microstate D time coverage (16). From this measure, a score value was computed (17) and smoothed using a linearly time-weighted average of the ten most recent samples (18). Finally, the feedback value was linearly scaled to the [–1, +1] range using the pre-estimated calibration extrema (18), and the corresponding visual feedback gauge was updated in real time (19).*

*
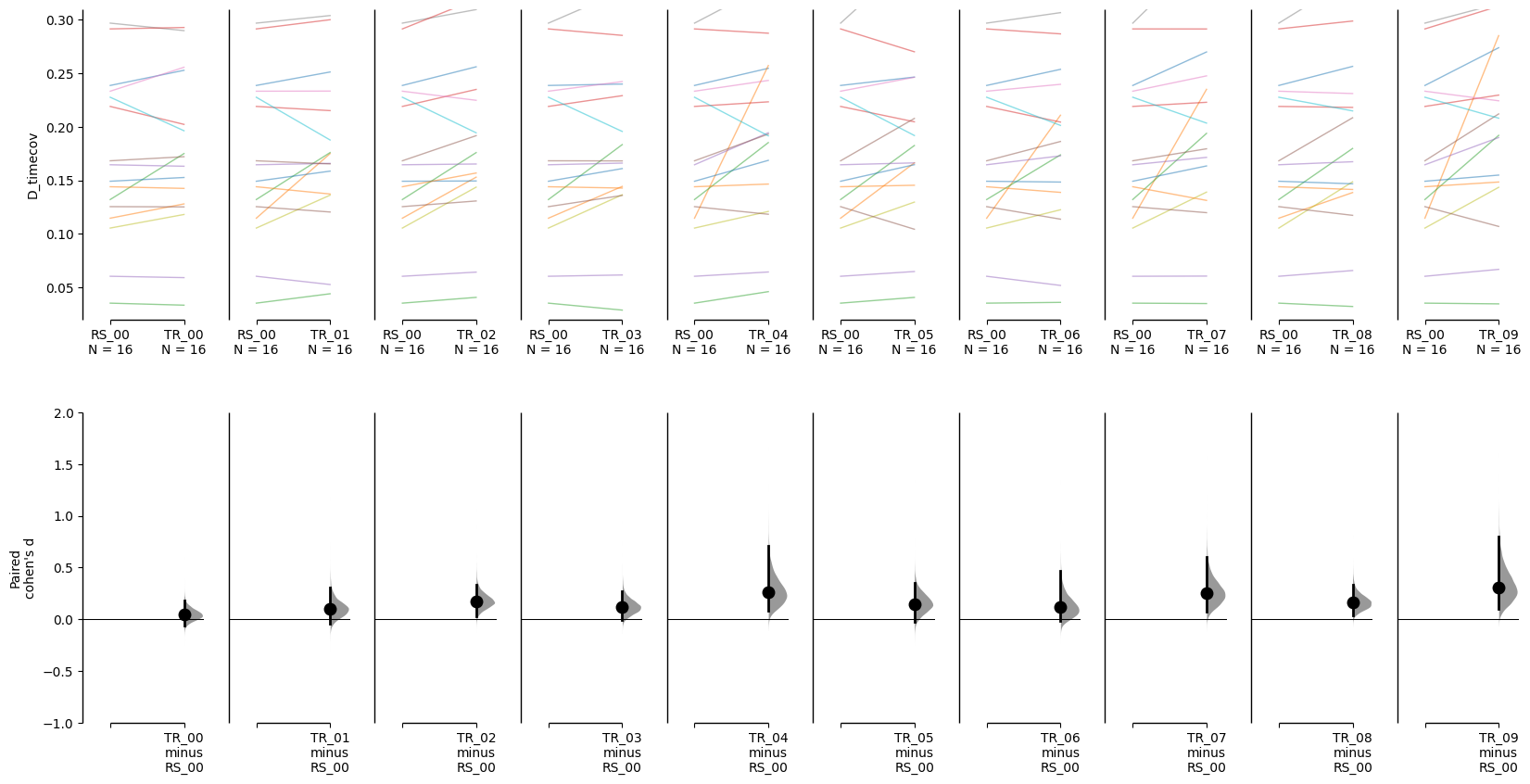
*

***Supplementary Fig 2****: Up regulation session: Estimation plots of paired Cohen’s d in microstate D time coverage between neurofeedback block and baseline during the up-regulation session. The first row represents the paired microstate D time coverage values between neurofeedback training block (TR) and baseline (RS*_0_*). Each line represents a participant. The second row displays the 95% confidence interval (95% CI) estimated through non- parametric bootstrap resampling of the paired Cohen’s d between each experimental conditions and baseline.*


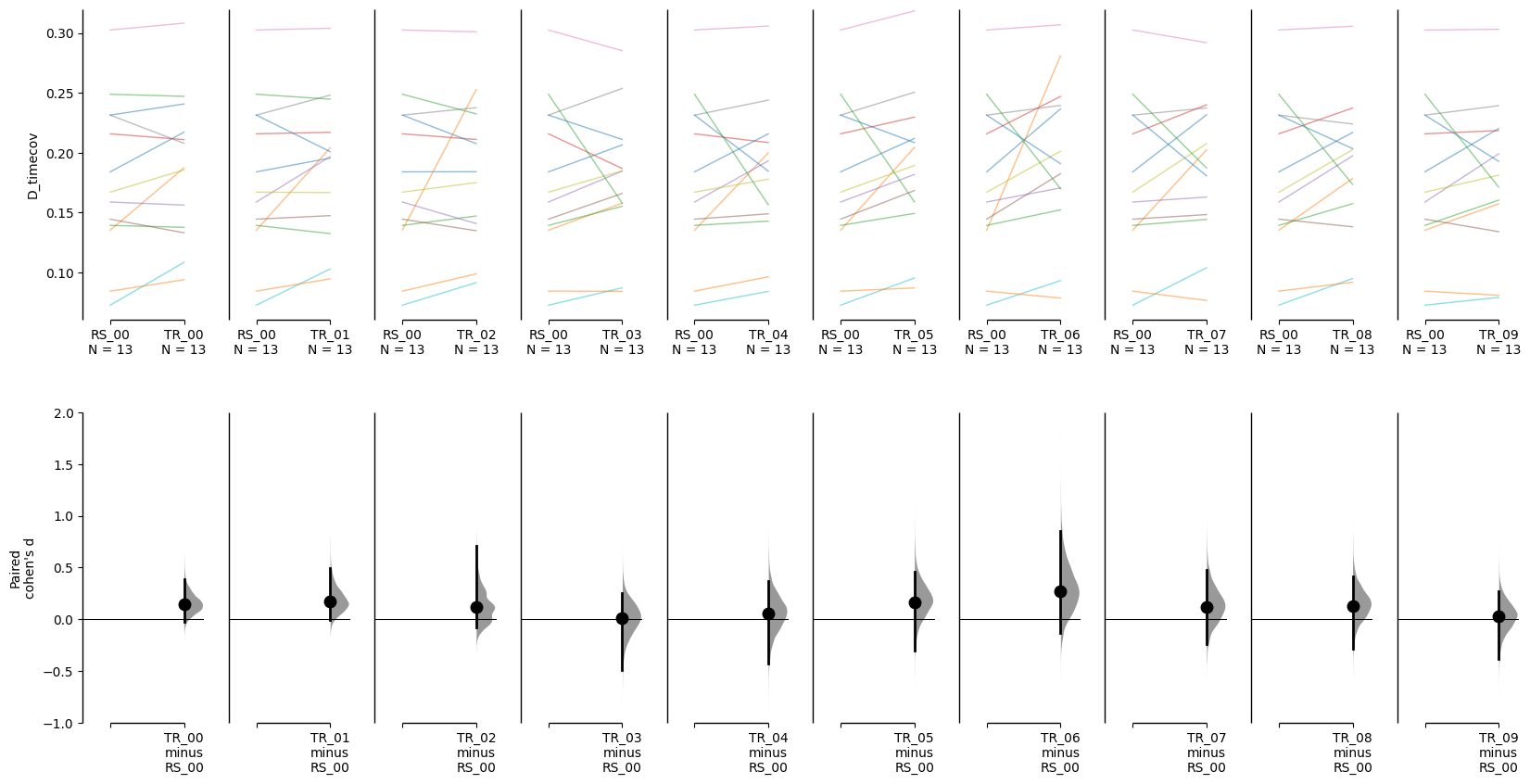


***Supplementary Fig 3****: Down regulation session: Estimation plots of paired Cohen’s d in microstate D time coverage between neurofeedback block and baseline during the up-regulation session. The first row represents the paired microstate D time coverage values between neurofeedback training block (TR) and baseline (RS*_0_*). Each line represents a participant. The second row displays the 95% confidence interval (95% CI) estimated through non- parametric bootstrap resampling of the paired Cohen’s d between each experimental conditions and baseline.*
